# MicroRNA-181a regulates IFN-γ expression in effector CD8^+^ T cell differentiation

**DOI:** 10.1007/s00109-019-01865-y

**Published:** 2020-01-30

**Authors:** Tiago Amado, Ana Amorim, Francisco J. Enguita, Paula V. Romero, Daniel Inácio, Marta Pires de Miranda, Samantha J. Winter, J. Pedro Simas, Andreas Krueger, Nina Schmolka, Bruno Silva-Santos, Anita Q. Gomes

**Affiliations:** 1 Instituto de Medicina Molecular João Lobo Antunes, Faculdade de Medicina, Universidade de Lisboa, Lisbon, Portugal; 2 Present Address: Institute of experimental Immunology, University of Zurich, Zurich, Switzerland; 3 Institute for Molecular Medicine, Goethe University Frankfurt, Frankfurt, Germany; 4 Present Address: Department of Molecular Mechanisms of Disease, University of Zurich, Zurich, Switzerland; 5 H&TRC Health & Technology Research Center, ESTeSL - Escola Superior de Tecnologia da Saúde, Instituto Politécnico de Lisboa, Lisbon, Portugal

**Keywords:** MicroRNA-181a, IFN-γ expression, CD8^+^ T cell

## Abstract

CD8^+^ T cells are key players in immunity against intracellular infections and tumors. The main cytokine associated with these protective responses is interferon-γ (IFN-γ), whose production is known to be regulated at the transcriptional level during CD8^+^ T cell differentiation. Here we found that microRNAs constitute a posttranscriptional brake to IFN-γ expression by CD8^+^ T cells, since the genetic interference with the Dicer processing machinery resulted in the overproduction of IFN-γ by both thymic and peripheral CD8^+^ T cells. Using a gene reporter mouse for IFN-γ locus activity, we compared the microRNA repertoires associated with the presence or absence of IFN-γ expression. This allowed us to identify a set of candidates, including miR-181a and miR-451, which were functionally tested in overexpression experiments using synthetic *mimics* in peripheral CD8^+^ T cell cultures. We found that miR-181a limits IFN-γ production by suppressing the expression of the transcription factor *Id2*, which in turn promotes the *Ifng* expression program. Importantly, upon MuHV-4 challenge, miR-181a-deficient mice showed a more vigorous IFN-γ^+^ CD8^+^ T cell response and were able to control viral infection significantly more efficiently than control mice. These data collectively establish a novel role for miR-181a in regulating IFN-γ–mediated effector CD8^+^ T cell responses in vitro and in vivo.

**Electronic supplementary material:**

The online version of this article (10.1007/s00109-019-01865-y) contains supplementary material, which is available to authorized users.

## Introduction

Interferon-γ (IFN-γ) is a critical cytokine in immunity against viral and intracellular bacterial infections as well as for tumor control. Studies with genetically modified mice lacking IFN-γ responses (with either *Ifng* or *Ifng* gene receptor 1 disruptions) have clearly shown a high susceptibility to bacteria, protozoans and viral infections [1]. Moreover, when challenged with chemical carcinogens, IFN-γ-deficient mice develop more tumors, and more rapidly than wild-type animals [2, 3].

CD8^+^ (herein simplified to CD8) T cells are a key source of IFN-γ within the adaptive immune response and play crucial roles in the control of intracellular infections and tumorigenesis [4, 5]. Consistent with this, studies enhancing the production of IFN-γ by CD8 T cells have shown improved antitumor responses in vivo in several mouse models of cancer [6, 7], and the robust activation of human CD8 T cells, including an IFN-γ molecular signature, are thought to underlie the recent successes of checkpoint inhibitors in cancer treatment [8].

After antigen recognition, activated CD8 T cells undergo proliferative expansion and differentiate into cytotoxic T lymphocytes (CTLs) that are able to produce effector molecules, among which IFN-γ and the cytotoxicity mediators perforin and granzyme B [4]. IFN-γ is the key orchestrator of the CTL response, since it not only boosts cytotoxicity but also upregulates the expression of MHC class I that is critical for antigen recognition and activation of CD8 T cells [1].

The induction of IFN-γ expression is a tightly regulated process in effector CD8 T cell differentiation. At steady state, naïve CD8 T cells produce little IFN-γ, but there is a marked upregulation upon TCR activation, with synergistic inputs from CD27 and CD28 coreceptors and interleukin- (IL-)12 and IL-18 signals [9, 10].

Downstream of cell surface signals, the process is controlled at the transcriptional level, where the transcription factors T-bet and Eomesodermin (Eomes) play the central roles [11, 12]. These seemingly play complementary roles in CD8 T cell differentiation, since T-bet expression associates with effector phenotype whereas Eomes levels increase in memory CD8 T cells [12].

Concomitant with major transcriptional changes, CD8 T cell differentiation has been recently associated with microRNA (miRNA)-mediated posttranscriptional regulation. Thus, while they are globally required for thymic CD8 T cell development [13, 14], miRNAs seemingly restrain cytotoxic effector CD8 T cell differentiation, as indicated by the increased perforin and granzyme B levels in mouse CD8 T cells genetically depleted of the miRNA processing enzyme, Dicer, and in human CD8 T cells where Dicer was knocked down by RNA interference [15]. Furthermore, various individual miRNAs have been identified either as positive or as negative regulators of CD8 T cell differentiation in vivo. For example, the downregulation of Let-7 (that targets Eomes and Myc mRNAs) promoted antiviral and antitumoral CD8 T cell responses [16]; and miR-23 blockade enhanced granzyme B expression in human CD8 T cells and inhibited tumor progression in a mouse model of cancer [17]. By contrast, miR-150-deficient mice showed poor cytotoxic effector functions and failed to respond to *Listeria* or viral infections [18]; and miR-155-deficient CD8 T cells were ineffective at controlling tumor growth and viral replication and clearance [19]. Conversely, miRNA-155 overexpression augmented the antitumor response in vivo [20], as well as the numbers of antiviral effector CTL, seemingly as consequence of enhanced T-bet expression, which is negatively regulated by a miR-155 target, SHIP-1 [21]. Moreover, miR-155 was shown to be essential to sustain exhausted CD8 T cell (Tex) responses during chronic viral infection by promoting the accumulation and persistence of Tex cells via Fosl2, an AP-1 transcription factor family member [22]. Contrarily, miR-31 promotes CD8 T cell dysfunction in chronic viral infection by increasing the sensitivity of T cells to type I interferons [23]. Some miRNAs also impact effector CD8 T cell proliferation and memory cell differentiation, as shown for the miR-17-92 cluster in the context of viral infection [24], whereas others bias CD8 T cell responses away from memory and toward effector CD8 T cell functions, as it is the case of miR-21, whose levels are associated with increased numbers of inflammatory effector T cells [25].

While these previous studies established the importance of various miRNAs in CD8 T cell (cytotoxic) responses in vivo, here we aimed at a focused dissection of miRNAs that may specifically regulate IFN-γ expression in CD8 T cells. Of note, other posttranscriptional mechanisms, including translational repression by RNA-binding proteins, have been shown to regulate IFN-γ expression by CD8 T cells [26]. As for miRNAs, while strongly implicated in the regulation of IFN-γ expression in CD4 T cells, most notably through miR-29 [27] and miR-125b [28], they remain poorly characterized in IFN-γ-producing CD8 T cells.

To address this issue, we screened miRNAs that segregated with IFN-γ expression ex vivo, in CD8 T cell subpopulations isolated from an IFN-γ reporter mouse model, and manipulated their activity in parallel in vitro and in vivo assays. This led us to identify miR-181a as a novel negative regulator of IFN-γ expression in CD8 T cells, whose absence enhanced CD8 T cells ability to respond and control viral infection in vivo.

## Methods

### Mice

All mice used were adults 6 to 12 weeks of age. C57BL/6J mice were purchased from the Jackson Laboratory. IFNγ-IRES-YFP-BGHpolyA knock-in mice (YETI) were purchased from Biocytogen. lckCre Dicer^Δ/Δ^ and Dicer^lox/lox^ mice were kindly provided by Matthias Merkenschlager, Imperial College, London [14]. miR-181a/b-1 ko mice (B6.*Mirc14*^tm1.1Ankr^) were described previously [29]. miR-451a ko mice were kindly provided by Lily Huang (UT Southwestern Medical Center, US).

Mice were bred and maintained in the specific pathogen–free animal facilities of Instituto de Medicina Molecular (Lisbon, Portugal). All experiments involving animals were done in compliance with the relevant laws and institutional guidelines and were approved by the ethics committee of Instituto de Medicina Molecular.

### Monoclonal antibodies

The following anti-mouse monoclonal antibodies (mAbs) were used (antigens and clones): fluorescently labeled anti-CD3 (145.2C11), anti-CD4 (GK1.5), anti-CD8 (53–6.7), anti-IFN-γ (XMG1.2), anti-IL-17A (TC11.18H10.1) purified anti-CD3 (145-2c11) and anti-CD28 (37.51).

Antibodies were purchased from BD Biosciences, eBiosciences, or BioLegend.

### Cell culture

Lymphocytes were cultured in RPMI medium supplemented with 10% FBS, 1% HEPES, 1% nonessential amino acids (NEAA), 1% sodium pyruvate (NaPu), 1% penicillin and streptomycin (Pen/Strep), 0.1% gentamicin, and 0.1% β-mercaptoethanol. Human embrionic kidney 293 T cells (CRL-3216, ATCC) were cultured in DMEM (high glucose, pyruvate) supplemented with 10% FBS. Baby hamster kidney fibroblast cells (BHK-21, ATCC) were cultured in Glasgow’s modified Eagle’s medium (GMEM) supplemented with 10% FBS, 2-mM glutamine, 100 U/ml penicillin, 100 g/ml streptomycin and 10% tryptose phosphate broth. All cells were incubated at 37 °C and 5% CO_2_.

### Cell preparation, flow cytometry, and cell sorting

Cell suspensions were obtained from spleens, lymph nodes, or thymus. Erythrocytes were osmotically lysed in red blood cell lysis buffer (BioLegend). Cells were filtered through 70-μm cell strainers (BD Biosciences). For cell surface staining, single-cell suspensions were incubated for 30 min with saturating concentrations of mAbs (see above). For intracellular cytokine staining, cells were stimulated with phorbol 12-myristate 13-acetate (PMA) (50 ng/ml), and ionomycin (1 μg/ml) in the presence of brefeldin A (10 μg/ml) (all from Sigma-Aldrich) for 3–4 h at 37 °C. Cells were stained for the above identified cell surface markers, fixed 30 min at 4 °C, permeabilized with the Foxp3 Transcription Factor Staining Buffer set (eBioscience) in the presence of anti-CD16/CD32 (eBioscience) for 15 min at 4 °C, and lastly incubated for 30 min–1 h at 4 °C with the above identified antibodies in permeabilization buffer. Samples were analyzed using LSRFortessa (BD Biosciences) and FlowJo software (Tree Star). For sorting, cells were prepared and stained for cell surface markers as mentioned above and sorted on a FACSAria (BD Biosciences).

### Redirect cytotoxic assay

YFP^+^ and YFP^−^ CD8^+^ T cells were sorted and were either cultured in complete RPMI for 72 h in a TPP 96 well U bottom plate, with plate-bound anti-CD3 mAb (1 μg/ml; 145.2C11; eBiosciences) and anti-CD28 mAb (1 μg/ml; 37.51; eBiosciences) or incubated directly after sorting in a 96 well U bottom plate for 4 h at 37 °C with P815 (ATCC® TIB-64™) mouse mastocytoma cell line, labeled with DDAOse (1 μM), with soluble anti-CD3 mAb (1 μg/ml; 145.2C11; eBiosciences). The ratio of T cell:P815 cell line ranged from 1:1, 1:5, or 1:10 as indicated in the respective figures. After 4 h of co-incubation, the cells were transferred to a 96 well V bottom plate and washed. Apoptosis was measured by staining with Alexa Fluor® 488 Annexin V/Dead Cell Apoptosis Kit (V13241; Life Technologies). Cells were resuspended and analyzed by flow cytometry on BD LSRFortessa cell analyzer.

### microRNA qPCR profiling

All experiments were conducted at Exiqon Services. The submitted RNA was reverse transcribed in 40 μl reactions using the miRCURY LNA Universal RT miRNA PCR, polyadenylation, and cDNA synthesis kit (Exiqon). cDNA was diluted 50 times and assayed in 10 μl PCR reactions according to the protocol for miRCURY LNA Universal RT miRNA PCR; each miRNA was assayed once by qPCR on the miRNA Ready-to-Use PCR, Rodent panel I. Negative controls, excluding template from the reverse transcription reaction, were performed and profiled like the samples. The amplification was performed in a LightCycler 480 real-time PCR system (Roche) in 384 well plates. The amplification curves were analyzed using the Roche LC software, both for determination of Cp (by the second derivative method) and for melting curve analysis. The amplification efficiency was calculated using algorithms similar to the LinReg software. All assays were inspected for distinct melting curves, and the Tm was checked to be within known specifications for the assay. Furthermore assays must be detected with five Cp’s less than the negative control and with Cp < 37 to be included in the data analysis. Data that did not pass these criteria were omitted from any further analysis. Using NormFinder the best normalizer was found to be the average of assays detected in all samples. All data was normalized to the average of assays detected in all samples (average – assay Cp).

The identification of potentially differentially expressed genes was performed by Exiqon based on a subtractive analysis. Briefly, for each miRNA, it calculated the average normalized expression value per group and the difference in expression between groups. It was then asked whether the inter-group difference was greater than 2 times the standard deviation of the YFP group for each particular miRNA.

### RNA isolation, complementary DNA production, and RT-qPCR

Total RNA (including mRNA and small RNA) was isolated using miRNeasy Mini Kit (Qiagen). mRNA was subsequently reverse transcribed with random oligonucleotides (Invitrogen) using Moloney murine leukemia virus reverse transcriptase (Promega). Extracted miRNA was subject to reverse transcription with miRCURY LNA Universal RT miRNA system (Exiqon). Both types of molecules were amplified by quantitative PCR on ViiA 7 real-time PCR system (Applied Biosystems; Life Technologies). mRNA primers were designed with Primer Blast or via the Universal Probe Library Assay Design Center (Roche) being their sequences included in Supplementary Table 1. miRNA LNA PCR primer sets were purchased from Exiqon. Analysis of quantitative PCR results was performed using the ViiA 7 software v1.2 (Applied Biosystems; Life Technologies). Results were normalized to the following reference genes: mir-423-3p or RNA RNU5G for miRNA quantification and Actb for mRNA quantification.

### CD8^+^ T cell electroporation

CD3^+^ CD8^+^ T cells were sorted from lymph nodes and spleens of C57BL/6 J mice and stimulated on a 24 well plate (300,000 cells per well) with plate-bound anti-CD3 and anti-CD28 (both at 2.5 μg/ml) for 48 h. Cells were then resuspended in T buffer and used (200,000 cells per condition) for transfection of mimics with the Neon electroporation transfection system (Invitrogen) using the 10 μl tip and applying the following parameters: 3 pulses of 10 ms and 1550 V. After electroporation, cells were cultured in 24 well plates with complete media without antibiotic supplemented with IL-2 (1 ng/ml) and 48 h later stimulated for intracellular cytokine staining. The mimics were used at 500 nM per electroporation. Mimics of miR-132-3p, miR-139-5p, miR-181a-5p, miR-200a-3p, miR-322-5p, and miR-451a, and negative control are all miRCURY LNA miRNA mimics from Exiqon. The siRNA against Id2, together with a nontargeting negative control, was obtained from Dharmacon and used at a concentration of 20–500 nM.

### Target gene prediction analysis

Predicted targets for mmu-miR-181a and mmu-miR-451a were determined by the DIANA micro-T [30], miRWalk 2.0 [31], miRanda [32], Targetscan [33], and RNA22 [34] algorithms. Validated targets for the same miRNAs were retrieved from miRTarBase [35].

### Luciferase assays

Plasmid vectors pMig-miR-181a and pMig-miR-451a that allow for the overexpression of miR-181a or mir-451a, respectively, were generated in the following manner: the respective native pre-miRNA sequences flanked by about 200 bp were amplified by PCR from genomic DNA (C57BL/6 J) and inserted into a modified pMig-IRES-GFP vector (Addgene #9044) [36]. The 3′UTR of Id2, Map2k1, and Akt was amplified by PCR from genomic DNA (C57 BL/6 J) and cloned into pmirGLO vector (Promega). Primers are available upon request. Mutations in the predicted target sequences of the 3′ UTR of Id2 were introduced by gene synthesis (GeneCust Europe). Each luciferase reporter vector carrying one of the 3’ UTR sequences described above was co-transfected with either the pMig-miR-181a or pMig-miR-451a expression vectors or a control empty pMig vector into HEK293 T cells (ATCC CRL-3216) using Lipofectamine 2000 (Thermo Fisher Scientific). After 48 h, firefly and *Renilla* luciferase activity were measured by using the Dual-Glo Luciferase Assay System (Promega). *Renilla* luciferase activity served as the internal control, and relative luciferase activity was normalized to empty pMirGlo and to empty pMig-IRES-GFP.

### Viral assays and infections

To prepare MuHV-4 (murid gammaherpesvirus-4) viral stocks, BHK-21 cells were infected at low multiplicity of infection (0.001 PFU per cell). Virions were recovered from debri-free supernatants by ultracentrifugation [37]. The titer of infectious virus was determined by plaque assay on BHK-21 cells. For in vivo infections, 6- to 8-week-old miR-181a/b-1^−/−^ and miR-451a^−/−^ mice and respective miR-181a/b-1^+/+^ and miR-451a^+/+^ C57BL/6 littermate controls were inoculated intranasally with 10^4^ PFU of MuHV-4 under isofluorane anesthesia. Lungs or spleens were harvested at the indicated time points. Titers of infectious virus were determined by plaque assay of freeze-thawed tissue homogenates on BHK-21 cells. Latent virus loads were quantified by explant co-culture of freshly isolated splenocytes with BHK-21 cells. Plates were incubated for 4 (plaque assays) or 5 (explant co-culture assays) days and then fixed with 4% formal saline and counterstained with toluidine blue for plaque counting.

### Statistical analysis

The statistical significance of differences between two populations was assessed using either the two-tailed nonparametric Mann-Whitney test or the t-test when applicable. ANOVA, followed by multiple comparisons, was performed when comparing the mean of more than one population. *P* values ≤ 0.05 were considered significant and are indicated in the figures. In bar graphs, data is presented as mean ± SD. In dot plots, horizontal lines indicate the mean.

## Results

### MicroRNA-deficient CD8 T cells overexpress IFN-γ

**Fig. 1:**
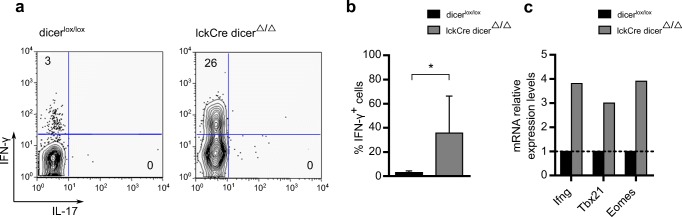
Dicer-deficient CD8^+^ thymocytes overexpress IFN-γ. (**a**) Representative plot (**a**) and quantification (**b**) of flow cytometry analysis of IFN-γ and IL-17 protein levels in thymic CD8^+^ T cells of dicer^lox/lox^ and lckCre dicer^Δ/Δ^ mice (*n* = 4) upon ex vivo stimulation with PMA and ionomycin for 4 h at 37 °C with the addition of Brefeldin A. (**c**) RT-qPCR analysis of *Ifng*, *Tbx21* and *Eomes* in thymic CD8^+^ T cells of dicer^lox/lox^ relative to lckCre dicer^Δ/Δ^ mice, normalized to housekeeping Actb mRNA. * *P* ≤ 0.05

To investigate the role of miRNAs in the regulation of IFN-γ expression in CD8 T cell differentiation, we considered that miRNAs could potentially inhibit this process. To test this hypothesis, we compared miRNA-deficient versus miRNA-sufficient CD8 thymocytes, since the latter should show very limited IFN-γ expression. Indeed, in stark contrast with minimal IFN-γ expression in Dicer-sufficient CD8 thymocytes, we found substantial IFN-γ-producing CD8 T cells in the thymus of a conditional Dicer-deficient mouse strain, controlled by the proximal *Lck* promoter, which therefore deleted Dicer at early stages of thymic T cell development [14] (Figs. 1a–b). More specifically, CD8 thymocytes from Dicer-deficient (lckCre Dicer^Δ/Δ^) mice contained almost 40% of IFN-γ-producing cells (upon short-term PMA plus ionomycin restimulation), compared to ~3% of IFN-γ^+^ CD8 thymocytes in Dicer-sufficient mice (Dicer^lox/lox^) (Figs. 1a–b). A less striking accumulation (< 10%) of IFN-γ^+^ cells was also observed in thymic CD4 T cells from Dicer-deficient mice when compared to controls (data not shown). Dicer-deficient CD8 T cells showed upregulated (when compared to Dicer-sufficient controls) mRNA levels of *Ifng* and its two main transcriptional regulators, *Tbx21* (encoding T-bet), and *Eomes* (Fig. 1c). These data revealed an unexpected and striking impact of the Dicer-dependent miRNA machinery on IFN-γ expression in thymic CD8 T cells and beckoned the identification of specific miRNAs underlying this phenomenon.

### Differential microRNA expression analysis identifies candidates segregating with IFN-γ expression in CD8 T cells

To identify individual miRNAs that might regulate IFN-γ expression in CD8 T cells, we compared the miRNome of cells either positive or negative for *Ifng* locus activity in the reporter mouse strain IFN-γ-IRES-YFP-BGHpolyA knock-in, also known as YETI (from Biocytogen). This model proved to be a suitable tool to study IFN-γ-producing CD8 T cells, given the association between YFP expression and intracellular IFN-γ staining (Supplementary Fig. 1a). Of note, we also observed enhanced cytotoxic potential of YFP^+^ (IFN-γ^+^) CD8 T cells when compared to their YFP^−^ (IFN-γ^−^) counterparts (Supplementary Fig. 1b), demonstrating the co-segregation of IFN-γ production with enhanced cytotoxic functions. This functional association, together with the expression pattern of the transcription factors, *Tbx21* and *Eomes* (Fig. 1c), demonstrate the global effector cell differentiation of IFN-γ^+^ CD8 T cells.

**Fig. 2:**
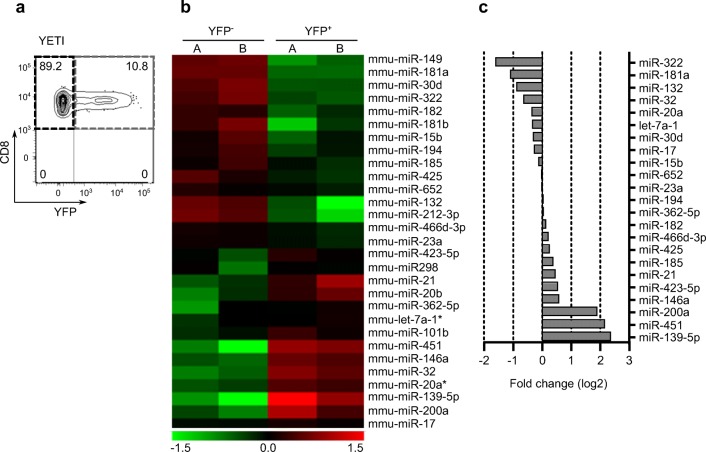
Differential expression analysis of microRNAs segregating with IFN-γ expression in CD8^+^ T cells. (**a**) Isolation by FACS of YFP^−^ vs YFP^+^ thymic CD8^+^ T cells from YETI mice for qPCR profiling. (**b**) Heatmap of differentially expressed genes between YFP^−^ and YFP^+^ CD8^+^ thymic T cells. The color scale shown at the bottom illustrates the relative expression level of a miRNA across all samples: red color represents an expression level above mean, and green color represents expression lower than the mean. (**c**) qPCR validation of selected miRNA genes’ differential expression between IFN-γ-producing vs nonproducing thymic CD8^+^ T cells. miRNAs enriched in YFP^−^ have negative log2 fold change values while miRNAs enriched in YFP^+^ have log2 fold change positive values

Upon FACS isolation of YFP^+^ (IFN-γ^+^) and YFP^−^ (IFN-γ^−^) CD8 T cells from the thymus of YETI mice (Fig. 2a), we extracted RNA, converted it to cDNA, and subjected it to qPCR profiling with miRCURY LNA™ Universal RT miRNA PCR Rodent panel I (Exiqon). From the 121 miRNAs identified in all samples, 29 were differentially expressed (at least twofold different) between YFP^+^ and YFP^−^ CD8 T cells, as depicted in the heatmap of Fig. 2b. Upon qPCR profiling validation with an independent sample (Fig. 2c), we selected top upregulated miRNAs in YFP^−^ CD8 T cells (miR-322, miR-181a, and miR-132) and top upregulated miRNAs in YFP^+^ CD8 T cells (miR-139, miR-451a, and miR-200) for subsequent functional studies.

### miR-181a and miR-451a limit IFN-γ production by CD8 T cells in vitro

**Fig. 3:**
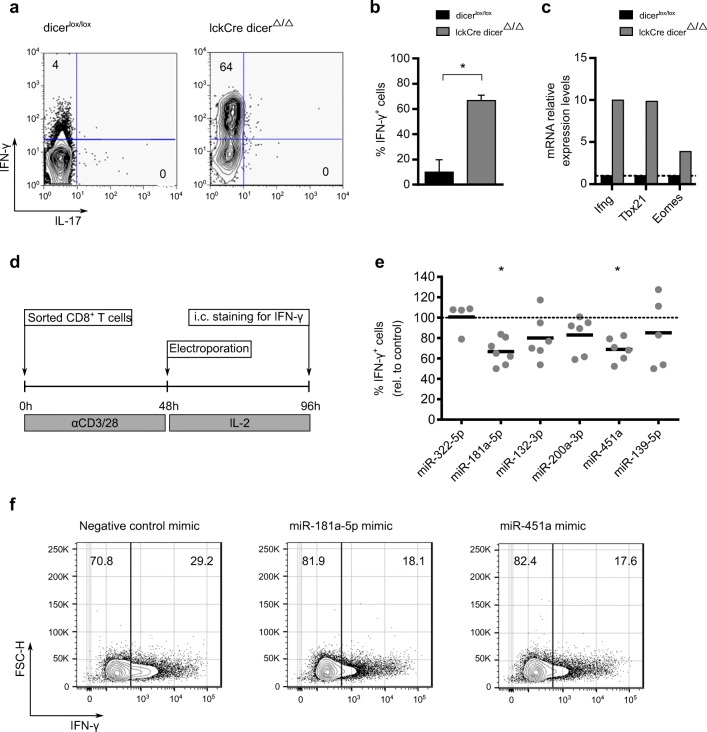
miR-181a-5p and miR-451a limit IFN-γ production by peripheral CD8^+^ T cells. Representative plot (**a**) and quantification (**b**) of flow cytometry analysis of IFN-γ and IL-17 protein levels in peripheral CD8^+^ T cells of dicer^lox/lox^ and lckCre dicer^Δ/Δ^ (*n* = 4). (**c**) RT-qPCR analysis of *Ifng*, *Tbx21* and *Eomes* in peripheral CD8^+^ T cells of dicer^lox/lox^ relative to lckCre dicer^Δ/Δ^ mice, normalized to housekeeping Actb mRNA. (**d**) Representation of the workflow followed for electroporation of miRNA mimics. Quantification (**e**) and representative plots (**f**) of flow cytometry analysis of IFN-*γ* production by CD8^+^ T cells after electroporation with indicated miRNA mimics (*n* = 5 to 7). Values in (**e**) are relative to those of each experiment control condition where a nontargeting negative control miRNA Mimic was transfected. * *P*≤ 0.05

To perform functional assays with candidate miRNAs, we turned to peripheral CD8 T cells. First, we assessed whether the IFN-γ phenotype observed in the Dicer-deficient thymus (Figs. 1a–c) was conserved in secondary lymphoid organs and could potentially contribute to CD8 T cell responses in the periphery. That was indeed the case: CD8 T cells isolated from pooled spleen and lymph nodes from lckCre Dicer^Δ/Δ^ mice showed markedly enhanced IFN-γ production (Figs. 3a–b) and *Tbx21* and *Eomes* expression (Fig. 3c), when compared to control (Dicer^lox/lox^) CD8 T cells.

We next performed overexpression studies by electroporating synthetic miRNA mimics in CD8 T cells isolated from pooled spleen and lymph nodes, a strategy successfully used in our lab before [36]. These were cultured for 2 days with plate-bound anti-CD28 and anti-CD3 monoclonal antibodies and subsequently electroporated with either control or specific mimics (Exiqon) for the six selected miRNAs (from Fig. 2c). After the electroporation, cells were cultured for two extra days in the presence of IL-2; and IFN-γ production was determined by intracellular staining after a short activation with PMA/ionomycin (Fig. 3d). We found that miR-181a and miR-451a, unlike the other miRNA candidates, significantly decreased IFN-γ production when compared to control mimics (Fig. 3e, f). These results identify miR-181a and miR-451a as potential new miRNA regulators of IFN-γ production by CD8 T cells. Interestingly, when we investigated what could induce miR-181a and miR-451a expression in CD8 T cells, we found signals that promote IFN-γ responses, such as the cytokines IL-12, IL-15, and IL-18 (Supplementary Fig. 2). This suggests that these miRNAs may act as an auto-regulatory mechanism during effector CD8 T cell differentiation, namely, miR-451a, which is overexpressed in IFN-γ^+^ (YFP^+^) CD8 T cells and is significantly upregulated by IFN-γ-promoting signals and could be responsible for fine tuning IFN-γ production in differentiated effector CD8 T cells. On the other hand, miR-181a (overexpressed in IFN-γ^−^ (YFP^-^) CD8 T cells) could be responsible for restricting CD8 T differentiation into IFN-γ−producing cells.

### miR-181a inhibits IFN-γ production by targeting *Id2*

**Fig. 4:**
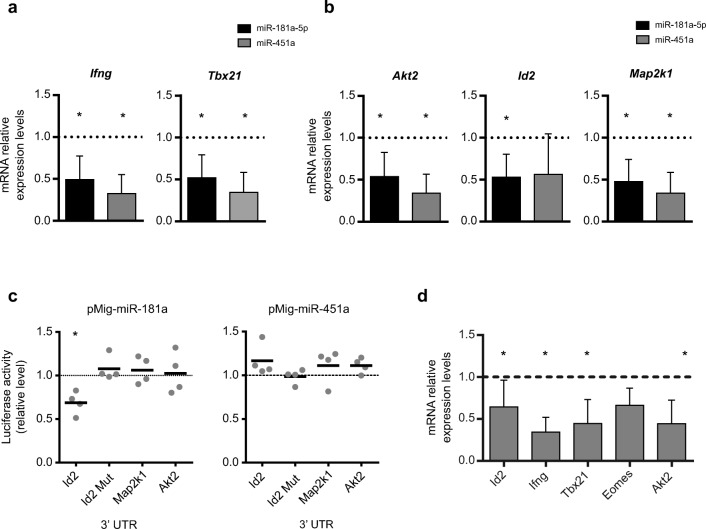
miR-181a-5p and its mRNA target *Id2* conversely regulate *Ifng* expression in CD8^+^ T cells. RT-qPCR analysis of (**a**) signature genes *Ifng* and *Tbx21* and (**b**) miR-181a-5p target genes *Akt2*, *Id2*, and *Map2k1* upon transfection of cells with either miR-181a-5p or miR-451a mimics (*n* = 4). Values are relative to those of each experiment control condition where a nontargeting negative control miRNA Mimic was transfected. (**c**) Luciferase reporter assay to verify interaction between overexpressed microRNAs, miR-181a or miR-451a, and 3’ UTRs of selected targets: Id2, Map2k1 or Akt2. Mutation of miR-181a-5p binding sites in Id2 3’ UTR followed by luciferase assay was performed to prove miR-181a-binding specificity. Renilla/luciferase ratios were normalized to those obtained for empty pMig construct (*n* = 4). (**d**) RT-qPCR analysis of *Id2*, *Ifng*, *Tbx21*, *Eomes*, and *Akt2* genes after siRNA-mediated Id2 knockdown (*n* = 4). Values are relative to those of each experiment control condition where a nontargeting negative control siRNA was transfected.* *P* ≤ 0.05

To understand how miR-181a and miR-451a could impact CD8 T cell differentiation and IFN-γ production, we next aimed at identifying their relevant mRNA targets in this process. We used bioinformatics to assess predicted and validated target mRNAs, thus containing binding sites for miR-181a-5p or miR-451a in their 3′ untranslated region (3’ UTR) (Supplementary Table 2). This analysis selected both canonical and noncanonical targets based on the evidence that noncanonical targets are functionally relevant as recently described in various seminal papers such as: in the work by Kim D and collaborators [38]. From these, we selected a set of genes, namely signaling molecules or transcription factors, based on their relevance for the IFN-γ expression program according to the literature (Fig. 4a–b and Supplementary Fig. 3a–b). To test whether their expression levels were modulated by miR-181a-5p or miR-451a, we electroporated the corresponding mimics in CD8 T cells (inducing approximately a tenfold upregulation of miRNA levels; data not shown) and subsequently performed RT-qPCR for the putative mRNA targets. Consistent with the IFN-γ protein data (Fig. 3e and f), the mRNA expression levels of both *Ifng* and its master transcription regulator *Tbx21* (T-bet) were reduced upon miR-181a-5p and miR-451a mimics transfection (Fig. 4a). Importantly, of the 11 potential direct mRNA targets of these miRNAs that we investigated (selected from validated and predicted targets of Supplementary Table 2: Akt2, Akt3, Mapk1, Id2, Map2k1, Map3k7, NFAT5, and Ptpn11 for miR-181a; Irf1, Akt3, Ankrd17, and Atf2 for miR-451a), only 3 were significantly downregulated: *Id2*, *Akt2*, and *Map2k1* (Fig. 4b and Supplementary Fig. 3a–b). These are important signaling components of the IFN-γ expression program: Akt2 and Map2k1 are signal transducing kinases, and Id2 is a key transcription factor in this process [39]. Interestingly, these three mRNAs have predicted binding sites for miR-181a (Supplementary Table 2) but not for miR-451a. Of note, our results defined specific signaling mediators of the IFN-γ program as being regulated by miR-181a, since, for example, *Akt2* but not *Akt3* and *Map2k1* but not *Mapk1* (encoding Erk) nor *Map3k7* were impacted in the overexpression experiments (Fig. 4b and Supplementary Fig. 3a–b).

To functionally validate the miRNA-mRNA interaction, we designed reporter constructs in a pmiRGlo Dual-luciferase miRNA target expression vector for the 3’ UTRs of *Id2*, *Map2k1*, and *Akt2*. These constructs were transiently transfected into human embryonic kidney (HEK) 293 cells together with an expression plasmid for either miR-181a or miR-451a. Co-transfection of miR-181a with these targets showed a significant repression of luciferase activity only for *Id2* (Fig. 4c); and mutations in the *Id2* putative binding sites to miR-181a led to a significant recovery of luciferase levels (Fig. 4c). These results are consistent with miRTarBase information that indicates *Id2* as a validated as a target for miR-181a [35, 40]. Although *Map2K1* and *Akt2* also had predicted binding sites for miR-181a, their reporter levels were not downregulated by ectopic expression of miR-181a, suggesting they may be indirectly regulated by the effects of miR-181a on other (unknown) targets. On the other hand, in agreement with the lack of predicted binding sites for miR-451a, none of the three 3’UTR regions interacted with miR-451a (Fig. 4c), also pointing toward alternative mechanisms of regulation. We therefore focused on the miRNA-mRNA interaction functionally validated with these assays, miR-181a: *Id2*.

Importantly, Id2 has been well established as an important regulator of CD8 T cell differentiation [41, 42] as well as IFN-γ expression [39]. We thus used RNA interference (siRNA) to downregulate (~40% relative to control levels) Id2 in peripheral CD8 T cells and observed a similar phenotype to the miR-181a overexpression (Fig. 4a–b), i.e., the reduction in *Ifng*, *Tbx21*, and *Akt2* levels (Fig. 4c). These data suggest that the miR-181a: Id2 pathway regulates IFN-γ expression in CD8 T cells in vitro.

### miR-181a limits antiviral CD8 T cell responses in vivo

**Fig. 5:**
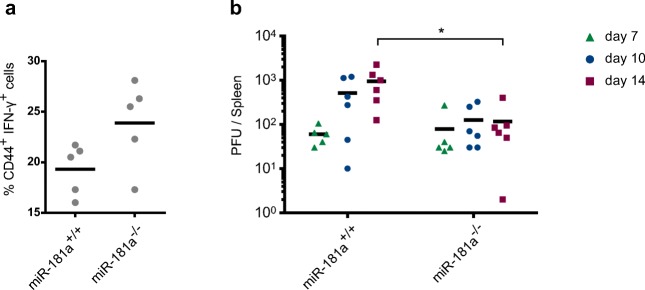
Lack of miR-181a-5p enhances antiviral CD8^+^ T cell responses in vivo. miR-181a^−/−^ and miR-181a^+/+^ littermates were infected intranasally with 10^4^ PFU of murid herpesvirus 4 (MuHV-4). (**a**) Percentages of activated (CD44^+^) CD8^+^ T cells expressing IFN-γ as assessed by intracellular staining. (**b**) Viral loads (PFU) quantified by ex vivo reactivation assays in which latently infected splenocytes (harvested at days 7, 10 and 14 postinfection) were co-cultured with permissive BHK-21 cells. Each dot represents one mouse. Horizontal lines indicate the mean. * *P* ≤ 0.05

Finally, to determine if miR-181a plays a nonredundant role in CD8 T cell function in vivo, we employed miR-181a^−/−^ mice (and miR-181a^+/+^ littermates) and challenged them with murid gammaherpesvirus 4 (MuHV-4) infection by intranasal inoculation. CD8 T cells harvested from spleens of miR-181a^−/−^ animals 7 days after infection produced higher levels of IFN-γ, compared to their miR-181a^+/+^ littermates (Fig. 5a). Consistently, miR-181a^−/−^ mice were more efficient in controlling the viral infection, as they presented lower viral loads in the spleen at subsequent time points (Fig. 5b), as determined by ex vivo reactivation assays in which latently infected splenocytes were co-cultured with permissive BHK-21 cells (Supplementary Fig. 4a). Of note, unlike miR-181a, miR-451a did not impact on viral load, nor on IFN-γ production by CD8 T cells, since miR-451a ^−/−^ mice showed similar responses to their miR-451a^+/+^ littermate controls (Supplementary Fig. 4b–d). These data further supported miR-181a as the most relevant miRNA, among the candidates retrieved by our study, for the regulation of effector CD8 T cell responses in vitro and in vivo.

## Discussion

Our study revealed that miRNAs constitute a key developmental brake to IFN-γ expression in CD8 T cells, since a large fraction (~40%) of Dicer-deficient CD8 thymocytes were able to produce the cytokine upon short-term restimulation, in stark contrast (~3%) to control (Dicer-sufficient cells) cells. This accumulation of IFN-γ-producing CD8 T cells in the thymus is reminiscent of previous reports on signaling mutant strains, including *Itk*-, *Klf2*-, *Cbp*-, and *Id3*-deficient mice, where CD8 T cells with memory-like phenotype and rapid IFN-γ responsiveness, in the absence of antigen exposure, have been termed “innate-like” and shown to participate in the early response against viral and intracellular bacterial infections [10, 43–46]. We therefore believe that miRNAs may act as a major brake to the development of such thymic-derived “innate-like” CD8 T cells, thereby promoting the adaptive mode of CD8 T cell response upon activation (and differentiation) in the periphery.

The impact of miRNAs on CD8 T cells clearly extends beyond IFN-γ expression. On one hand, the deletion of Dicer using the distal *Lck* promoter, which drives Cre expression after the stage of positive selection, resulted in robust responses to activation in vitro but has the incapacity to sustain survival and accumulation in vivo upon acute infection [47]. On the other hand, the deletion of Dicer in activated CD8 T cells caused a significant upregulation of the killing mediators, perforin and granzymes [15]. In the latter report, the specific miRNAs linked to the effector CD8 T cell phenotype were miR-139 and miR-150, which are distinct from the miRNA identified in our study as a key regulator of IFN-γ expression, miR-181a. This was identified as significantly enriched in CD8 T cells lacking IFN-γ expression (compared to IFN-γ^+^ counterparts) in YETI reporter mice. Of note, since YETI has modifications in the 3’ UTR of the *Ifng* mRNA, further functional analyses were performed in C57BL/6 WT mice to avoid artifacts in miRNA-mediated *Ifng* regulation.

We found miR-181a to be prominently expressed in thymic IFN-γ^−^ (YFP^−^) CD8 T cells and could therefore constitute a brake to IFN-γ induction in undifferentiated CD8 T cells. Consistent with this, using luciferase assays, we validated *Id2* as a miR-181a target and demonstrated that both Id2 downregulation and miR-181a (mimics) transduction reduced IFN-γ mRNA expression in effector CD8 T cells. Id2 is a key regulator of effector CD8 T cell differentiation and maintenance in vivo [41, 42] and has also been shown to promote the differentiation of IFN-γ-producing CD4^+^ T (so-called Th1) cells upon viral infection [39]. There is a clear mechanistic link between Id2 and IFN-γ, since Id2 directly antagonizes the suppressive E proteins that bind to regulatory elements of *Tbx21*, which encodes the master transcription factor T-bet [39]. Our results with Id2 knockdown, which very nicely phenocopied those of miR-181a mimics, constituted a critical validation of our experimental approach.

Importantly, our in vivo studies demonstrated that miR-181a is a key nonredundant determinant of antiviral IFN-γ^+^ CD8 T cell responses, with miR-181-deficient mice controlling MuHV-4 herpesvirus load significantly better than miR-181-sufficient littermate controls.

The new function disclosed here for miR-181a in the regulation of IFN-γ production and effector CD8 T cell responses in vivo adds to its established role in thymic positive selection, where it modulates TCR signaling toward augmenting thymocyte sensitivity to peptide antigens [48]. In vivo*,* the role of miR-181a is complex and only partially understood in positive and negative selection of conventional T cells [49, 50]. In contrast, miR-181a has turned out to be a critical mediator of agonist selection of unconventional T cells, including NKT cells, MAIT cells, and Treg cells [29, 51, 52]. Interestingly, this known role of miR-181a also involved the modulation of signaling components [48], which supports this type of mechanism as characteristic of miR-181a function in T cells. miR-181a induction by signals that promote IFN-γ responses, namely, TCR stimulation and the cytokines IL-12, IL-15, and IL-18, suggests that this miRNA can act as an auto-regulatory mechanism during effector CD8 T cell differentiation.

The results obtained for miR-451a are also indicative of a negative feedback loop since this miRNA is enriched in IFN-γ^+^ cells, and its ectopic overexpression reduces the percentage of IFN-γ^+^ cells. However, we failed to identify relevant mRNA targets for miR-451a. Furthermore, the lack of a nonredundant role in vivo when we compared the antiviral IFN-γ^+^ CD8 T cell response in miR-451a^−/−^ mice with that of miR-451a^+/+^ littermate controls further highlights the need for additional studies to dissect the potential role of miR-451a in CD8 T cell differentiation.

From a therapeutic point of view, the manipulation of miRNAs may offer the possibility to modulate IFN-γ responses by human CD8 T cells, consistent with previous findings using RNA interference-mediated knockdown of Dicer [15]. Specifically for miR-181a, its therapeutic potential is further supported by reported successful manipulation in human cells [53]. We thus believe that this study adds significantly to our understanding of the posttranscriptional regulation of IFN-γ production by CD8 T cells, which, in addition to the clear impact on antiviral responses in mice, may have implications in other contexts such as antitumor immunity or the control of IFN-γ–mediated inflammatory conditions.

## Electronic supplementary material

ESM 1 (PDF 3576 kb).

ESM 2 (XLSX 189 kb).
