# Supplementary material for: MicroRNA-181a regulates IFN-γ expression in effector CD8+ T cell differentiation
Source: J Mol Med (Berl). 2020 Jan 30;98(2):309–20. doi: 10.1007/s00109-019-01865-y (PMC7007887; doi:10.1007/s00109-019-01865-y)
Supplement: Supplementary file 1 — (PDF 3576 kb). [file 109_2019_1865_MOESM1_ESM.pdf]

Supplementary Figures

a

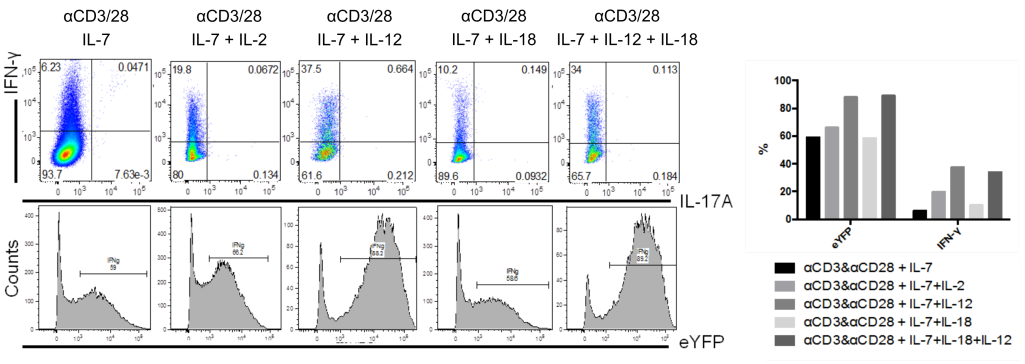

b

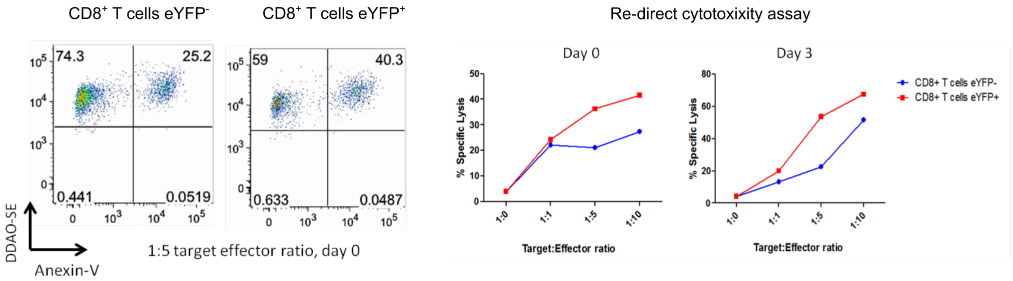

Supplementary Figure 1. CD8+ T cell effector functions segregate with YPF+ *versus* YFP- subsets of YETI reporter mice

(**a**) eYFP expression associates with intracellular expression of IFN-γ in Yeti mice, upon IFN-γ inducing conditions. Representative intracellular staining for IFN-γ and IL-17A in thymic CD8+ T cells of *Ifng*-YFP mice stimulated *in vitro* for four days with plate bound anti-CD3 and anti-CD28 and in the presence of different cytokines (IL-7, IL-2, IL-4, IL-12 and IL-18) (left, top). Representative histograms indicating the eYFP expression in the same conditions (left, bottom). Frequency of IFN-γ+ and YFP+ thymic CD8+ T cells (right). (**b**) Representative flow cytometry plots of Annexin-V staining at day 0 and with 1:5 target:effector ratio (left) and percentage of specific lysis (right) of the redirected lysis assay with (DDAO-SE-labelled) mastocytoma P815 target cell line by CD8+ eYFP+ cells and CD8+ eYFP- at day 0 and day 3.

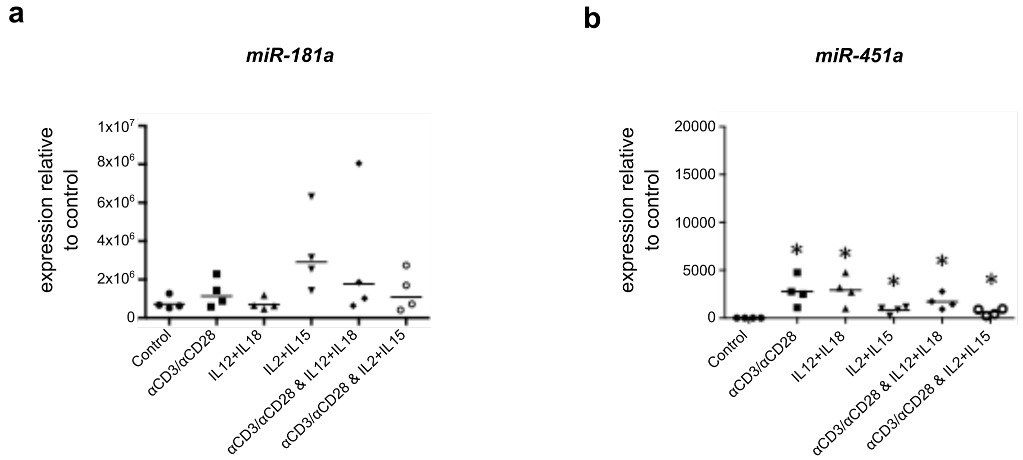

Supplementary Figure 2. miR181a and miR451a are upregulated by signals that boost IFN-γ responses. RT-qPCR analysis of (**a**) miR-181a, (**b**) miR-451, expression in peripheral $CD8^+$ T cells under various IFN-γ promoting culture conditions. Peripheral $CD8^+$ T cells were stimulated over night with IL-12 plus IL-18 or IL-2 plus IL-15 with or without TCR activation (plate bound anti-CD3 and anti-CD28). The conditions are indicated below each graph. Expression levels are relative to the reference miRNA miR-423-3p. (n=4). *P ≤ 0.05.

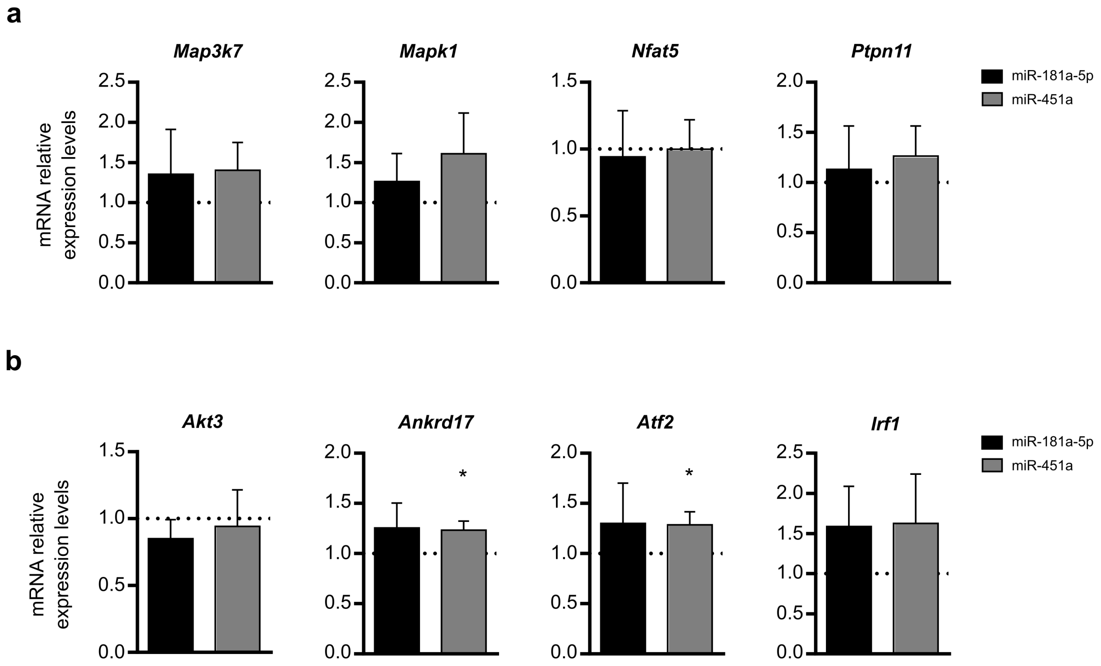

Supplementary Figure 3. Putative mRNA targets not impacted by miR-181a or miR-451a mimics transfection into CD8 T cells. RT-qPCR analysis of miR-181a-5p (**a**) or miR-451a (**b**) putative target genes upon transfection of cells with either miR181a-5p or miR-451a mimics (n=4). Akt3 is predicted to be a target of both microRNAs. * P ≤ 0.05

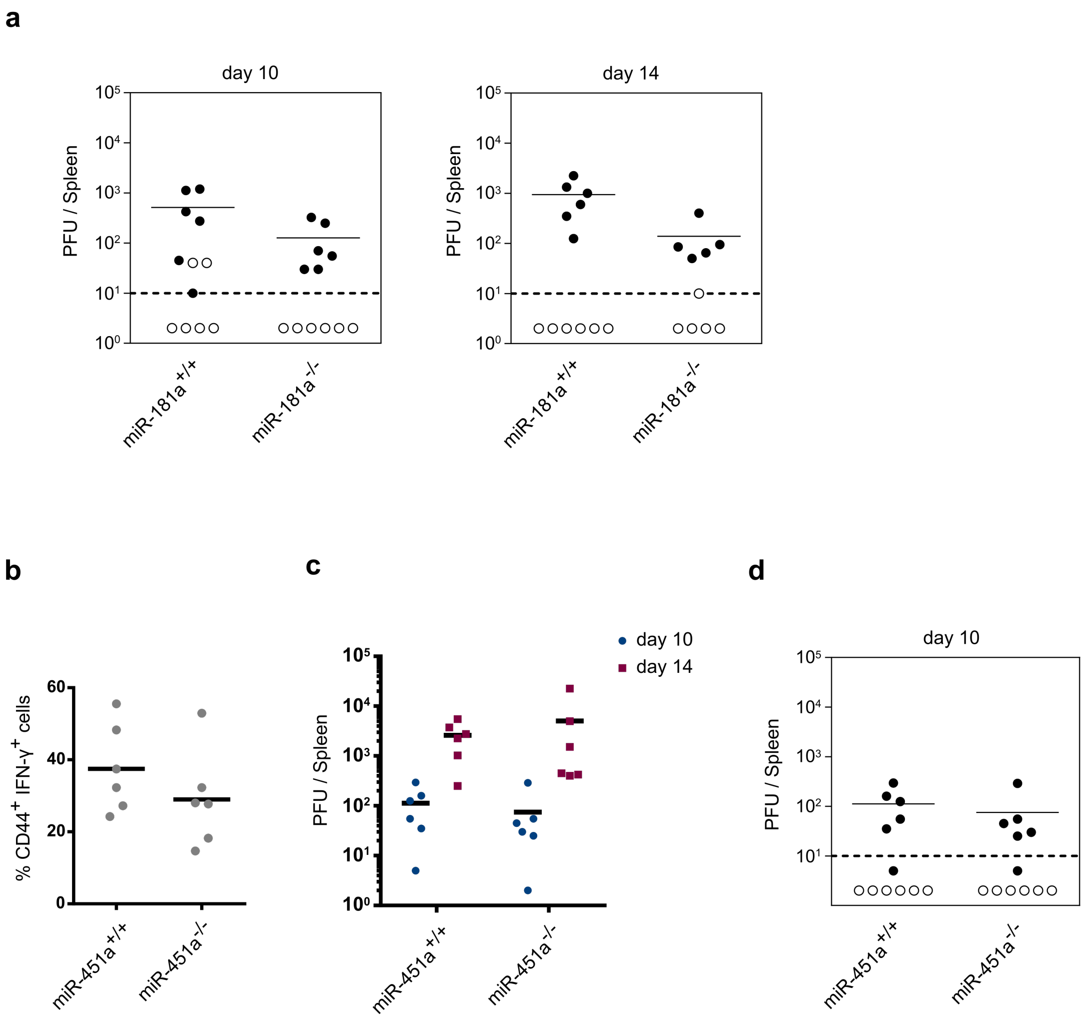

Supplementary Figure 4. Lack of miR-451a, contrary to lack of miR-181-5p, has no effect on anti-viral CD8^{+} T cell responses *in vivo*

(**a**) Viral titers in spleens of miR-181a$^{-/-}$ and miR-181a-5p$^{+/+}$ littermate mice infected with MuHV-4 of mice at 10 and 14 days post-infection. Black symbols, reactivating virus titers; open symbols, preformed infectious virus titers. Circles represent individual mice. Horizontal lines indicate the mean. The dashed line indicates the limit of detection.

(**b-d**) miR-451a$^{-/-}$ and miR-451a$^{+/+}$ littermate mice were infected intranasally with 10^4 PFU of MuHV-4. (**b**) Percentages of activated (CD44^{+}) CD8^{+} T cells expressing IFN-γ as assessed by intracellular staining. (**c**) Viral loads (PFU) quantified by *ex vivo* reactivation assays in which latently infected splenocytes (harvested at 10 and 14 days post-infection) were co-cultured with permissive BHK-21 cells to allow quantification of the plate forming units (PFU) present. (**d**) Viral titers in spleens of miR-451a$^{-/-}$ and miR-451a$^{+/+}$ littermate mice infected with MuHV-4 of mice at 10 days post-infection. Black symbols, reactivating virus titers; open symbols, preformed infectious virus titers. Circles represent individual mice. Horizontal lines indicate the mean. The dashed line indicates the limit of detection.

Supplementary Table 1. Sequence of primers used in RT-qPCR analysis

Gene	Primer forward (5'-3')	Primer Reverse (5'-3')
Actb	CGTGAAAAGATGACCCAGATCA	TGGTACGACCAGAGGCATACAG
Akt2	ATACCAGGCACCCCTTCCT	CACAAAGCATAGGCGGTCA
Akt3	GTGGACCACTGTTATAGAGAGAACAT	TTGGATAGCTTCCGTCCACT
Ankrd17	CTAAGCGTGGGCCAAAGA	TGGATATCACAGTTGATGGAACA
Atf2	GAAGAGTCTCGCCCACAGTC	TGGGTCTGAGGAGTTGTGTG
Eomes	CGTTCACCCAGAATCTCCTAACA	TGCAGCCTCGGTTGGTATTT
Id2	CGCATCCCACTATCGTCAG	AGCTCAGAAGGGAATTCAGATG
IFNγ	TCTTCTTGGATATCTGGAGGAACTG	GAGATAATCTGGCTCTGCAGGATT
Irf1	CACTGATCTGTATAACCTACAGGTGTC	CCTTCCTCATCCTCGTCTGT
Map2K1	CCATTCCTCCTCCTGATGC	GTCGGCTGTCCATTCCATA
Map3k7	CCATCCCAATGGCGTATC	TGCCATGGATTCTTTGGAGT
Mapk1	GACAGAGTACGTAGCCACACGTT	AGCCCACAGACCAAATATCAAT
Nfat5	CTCGGACTTCATCTCATTGCT	TCTGTGATGGGTGTAACTTCAGA
Ptpn11	AGCTGGCTGAGACCACAGAT	TGTTGCTGGAGCGTCTCA
Tbx21	CACACACGTCTTTACTTTCCAAGAGA	CACTCGTATCAACAGATGCGTACAT

Supplementary Table 2. miR-181a-5p and miR-451a validated and predicted targets

Note: Supplementary Table 2 is in a separate file as it has a large amount of information.
